# Supplementary material for: The negative emission potential of alkaline materials
Source: Nat Commun. 2019 Mar 28;10:1401. doi: 10.1038/s41467-019-09475-5 (PMC6438983; doi:10.1038/s41467-019-09475-5)
Supplement: Supplementary file 1 — Supplementary Information [file 41467_2019_9475_MOESM1_ESM.pdf]

Supplementary Information

The negative emission potential of alkaline materials

Phil Renforth

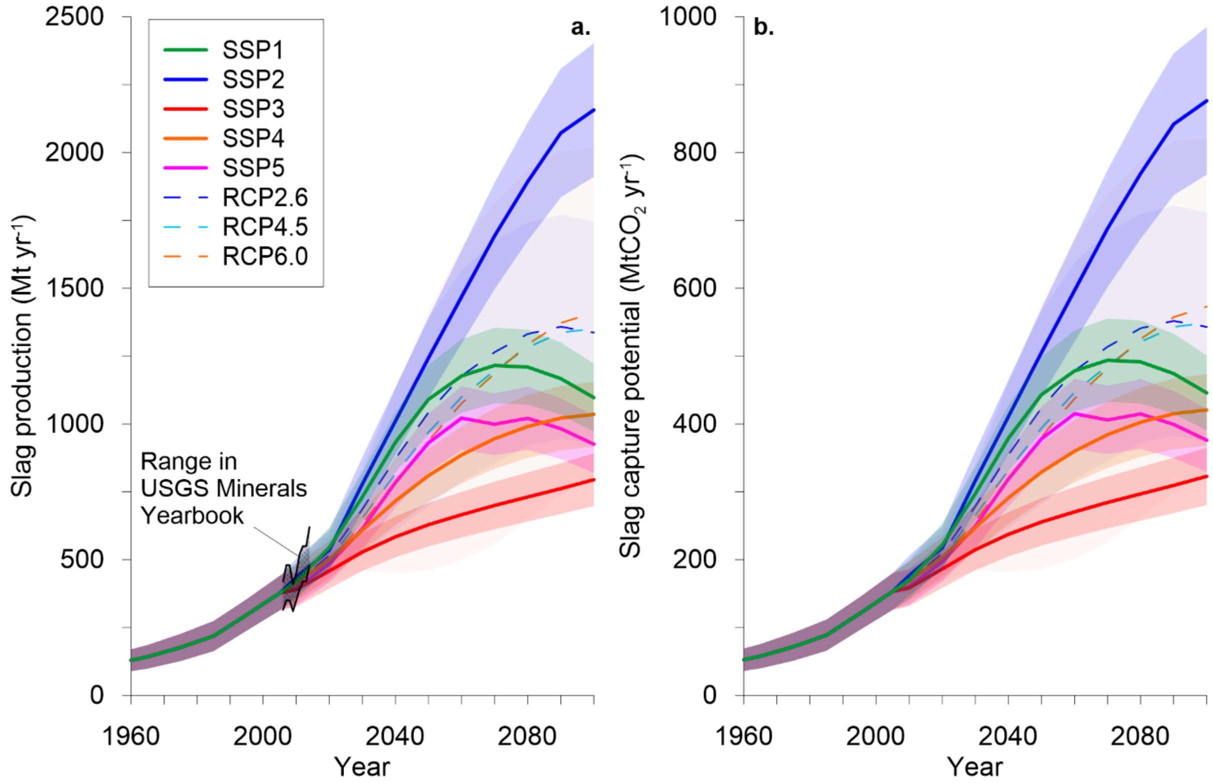

Supplementary Figure 1. Production forecasts for the iron and steel industry a. Global production of slag including estimates from the USGS [1], and **b.** the carbonation CO_2 capture potential. Error bars represent the standard error from the range of pathways (for SSP1,3 RCP2.6 and RCP 6.0 n = 4, for all others n = 5)

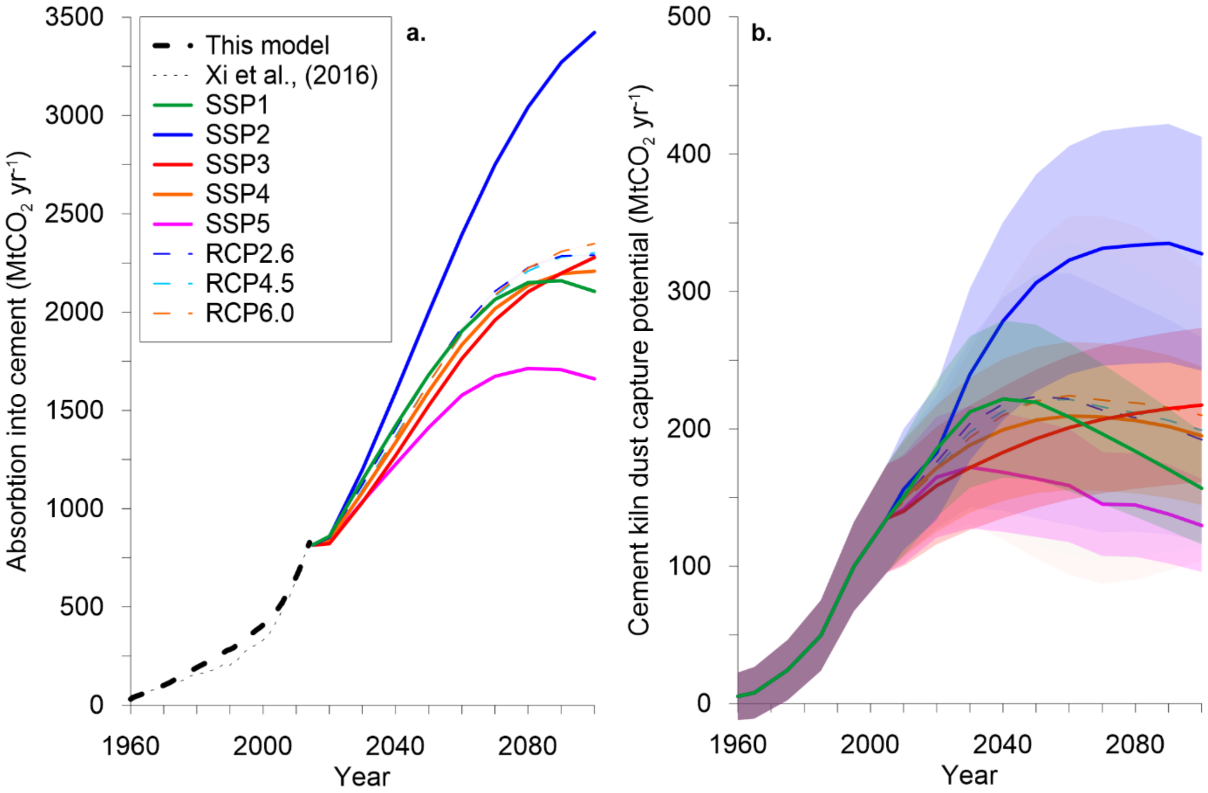

Supplementary Figure 2. Production forecasts for the cement industry a. The predicted absorption into cement (concrete and mortar) during service life and following demolition including the estimate from Xi et al.,[2] for comparison, and **b.** the CO_2 capture potential of cement kiln dust. Error bars represent the standard error from the range of pathways (for SSP1,3 RCP2.6 and RCP 6.0 n = 4, for all others n = 5)

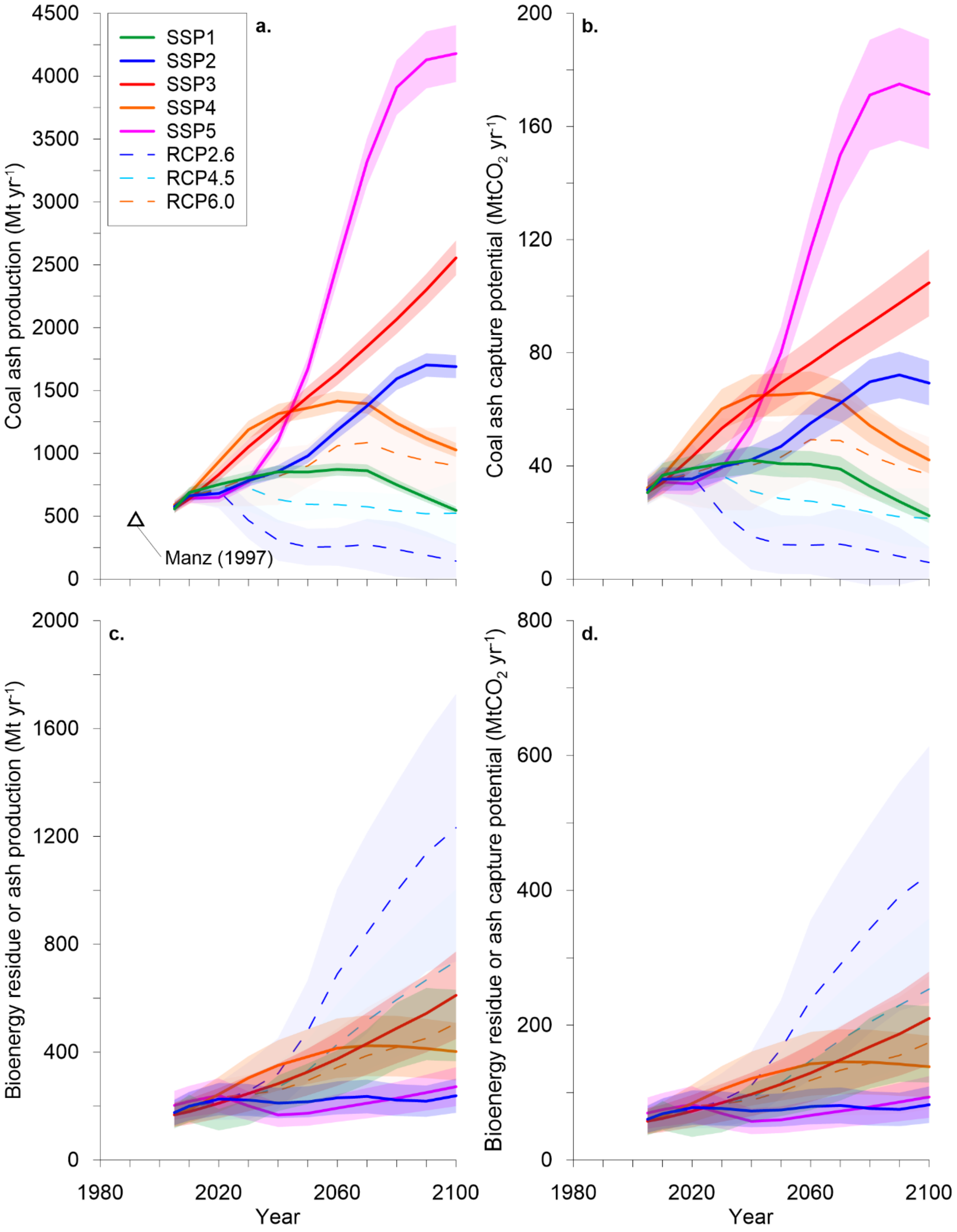

Supplementary Figure 3 Production forecasts for bioenergy a. Total coal ash production (including from hard coal and lignite) including an estimate of contemporary production in the early 1990's by Manz [3], and **b.** its associate carbonation potential. **c.** Total biomass energy ash/residue production, and **d.** its carbonation potential. Error bars represent the standard error from the range of pathways (for SSP1,3 RCP2.6 and RCP 6.0 n = 4, for all others n = 5)

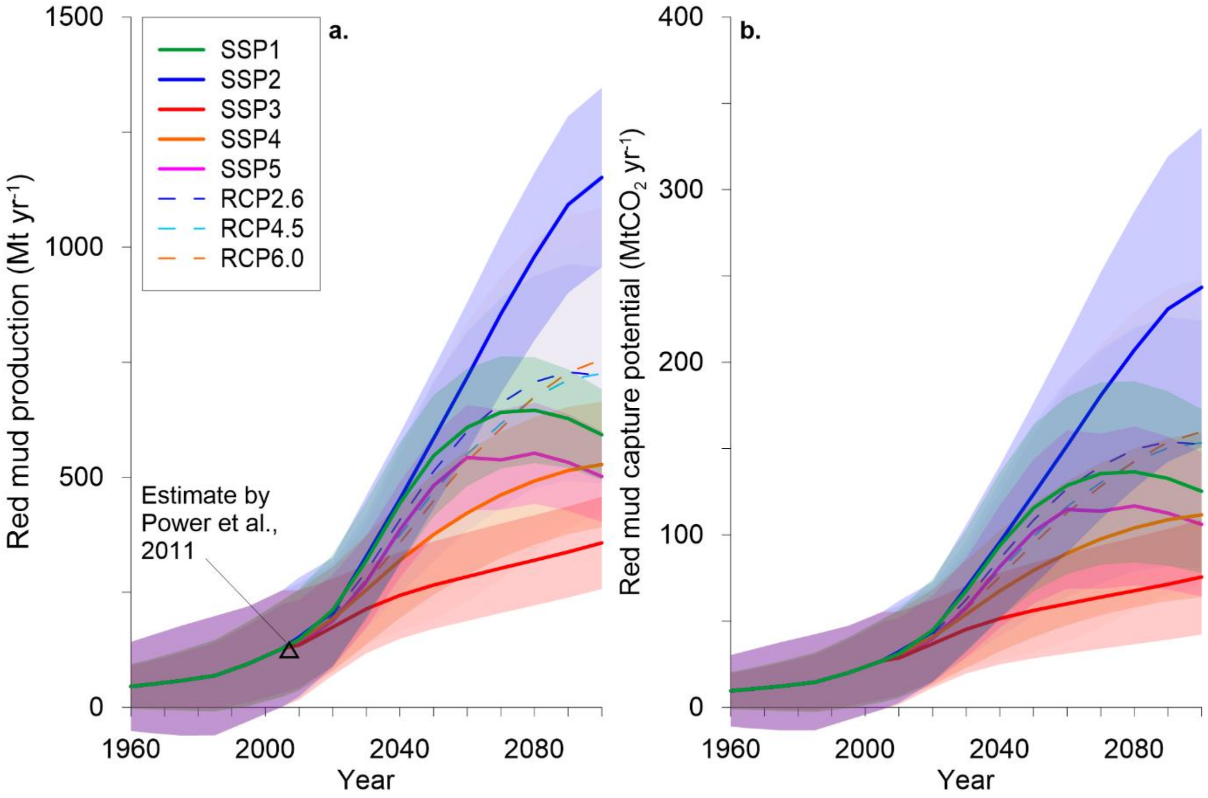

Supplementary Figure 4 Production forecasts for the aluminium industry a. Red mud production estimates including an estimate of contemporary production in 2007 by Power et al., [4], and **b.** the carbonation potential of red mud. Error bars represent the standard error from the range of pathways (for SSP1,3 RCP2.6 and RCP 6.0 n = 4, for all others n = 5)

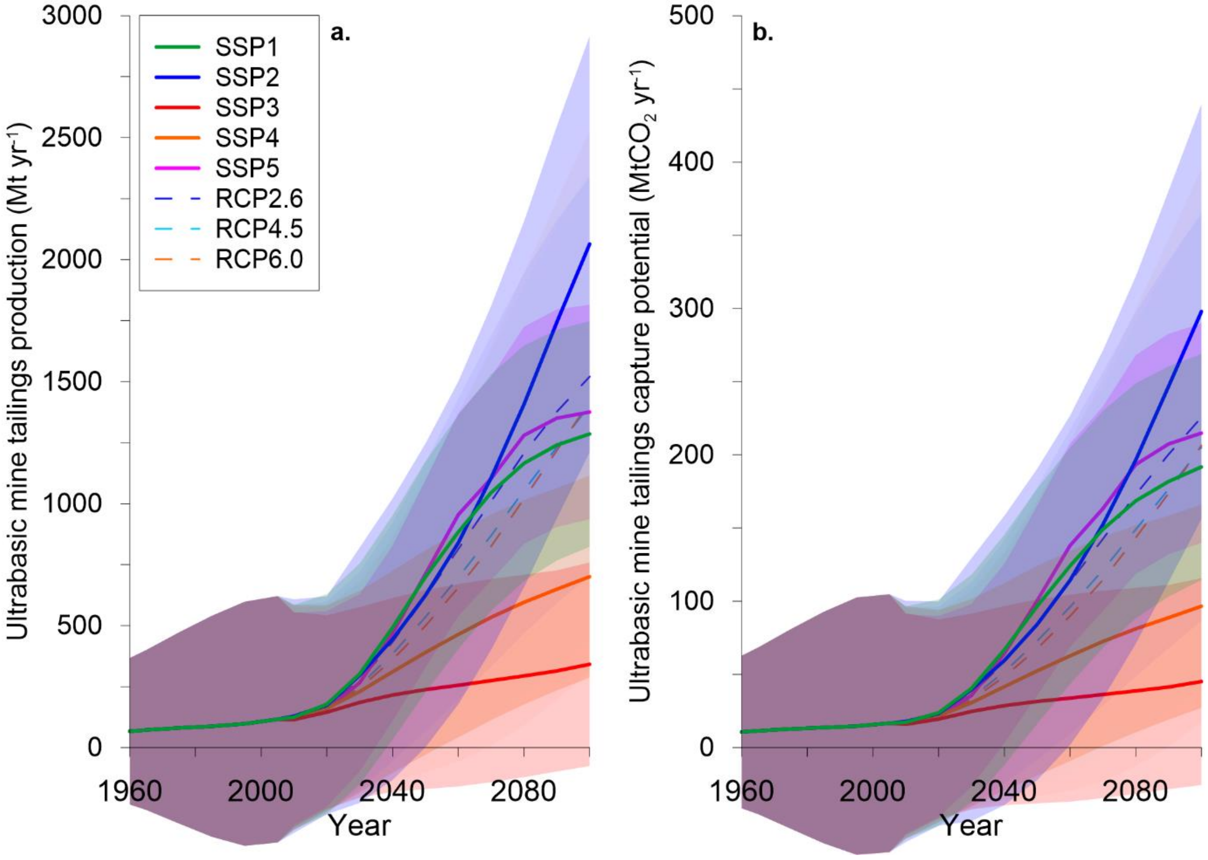

Supplementary Figure 5 Production forecasts for metal mining a. Ultrabasic mine tailings production estimates based on Ni laterite, Ni sulphide and PGM production. **b.** the carbonation potential of mine tailings. Error bars represent the standard error from the range of pathways (for SSP1,3 RCP2.6 and RCP 6.0 n = 4, for all others n = 5)

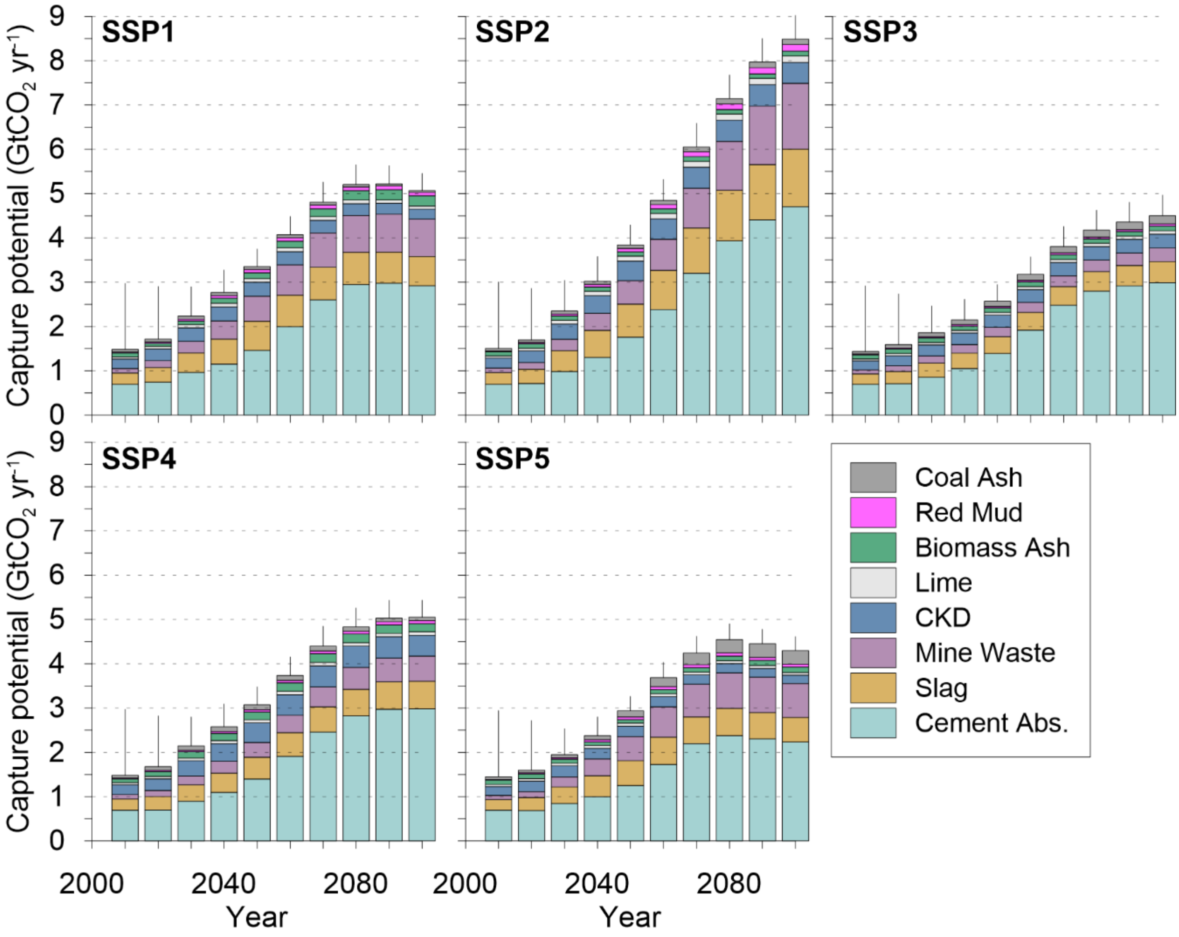

Supplementary Figure 6 CO_2 capture potential through enhanced weathering The various baseline Shared Socioeconomic Pathways (SSPS) are shown for the range of alkaline materials to 2100. Error bars represent the standard error from the range of pathways (for SSP1,3 n = 4, for all others n = 5)

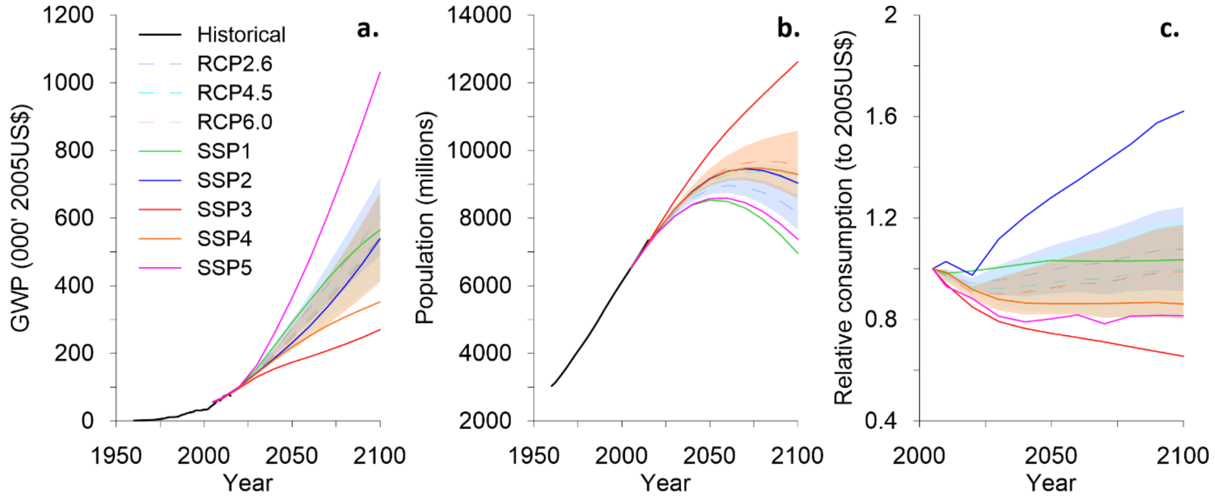

Supplementary Figure 7 Summary of economic and population projections a. future gross world product (GWP, PPP), **b.** population, **c.** and relative consumption used for driving the changes in material production. Error bars represent the standard error from the range of pathways (for SSP1,3 RCP2.6 and RCP 6.0 n = 4, for all others n = 5). The framework for Shared Socioeconomic Pathways (SSPs) and Representative Concentration Pathways (RCPs) [5], forecast future economic growth, population, and consumption were downloaded through the International Institute for Applied Systems Analysis web portal (https://tntcat.iiasa.ac.at/SspDb). Narratives associated with each future scenario are presented in [6], but are summarised as 'sustainability' (SSP1), 'middle of the road' (SSP2), 'regional rivalry' (SSP3), 'inequality' (SSP4), and 'fossil-fuelled development' (SSP5). The framework pairs baseline SSPs with RCPs [5]. Here we evaluate the alkaline material production potential of each of these SSPs and the mean value of the associated RCPs.

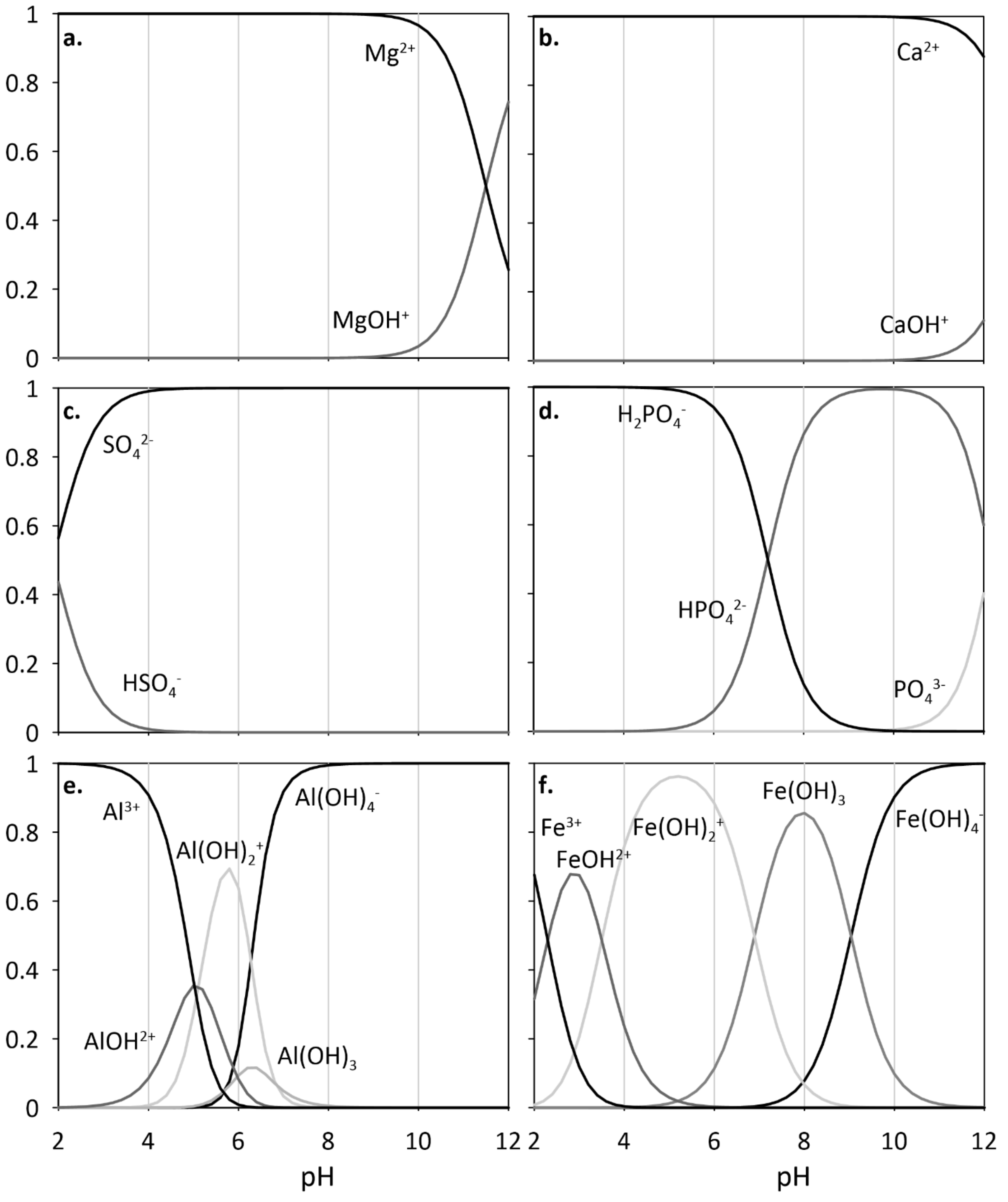

Supplementary Figure 8 Aqueous species of elements that may react with CO_2 a. magnesium, **b.** calcium, **c.** sulphur, **d.** phosphorus, **e.** aluminium, and **f.** iron. The pH dependent distributions at 25°C are shown in Supplementary Figure 2, which was generated using the geochemical modelling software PHREEQC v2 [7] and the associated the database file phreeqc.dat at standard temperature and pressure. The distribution of aqueous species of iron was further constrained by assuming the redox conditions within the solution were influenced by contact with atmospheric O_2 in which $\sim pe = 19 - pH$.

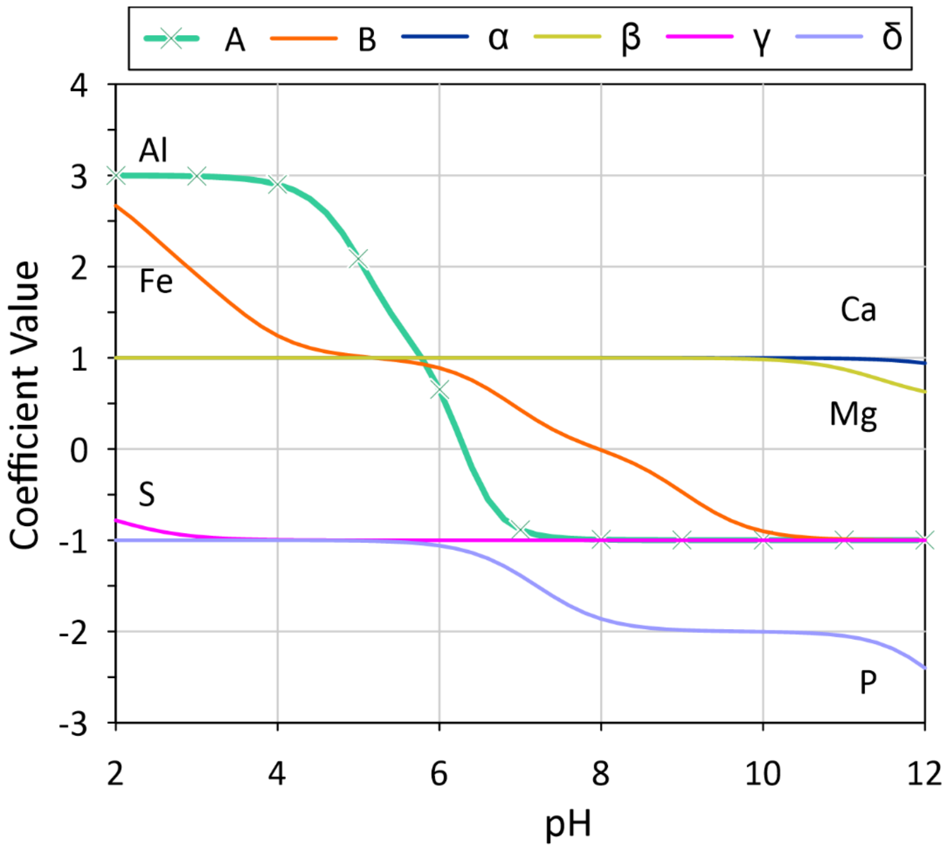

Supplementary Figure 9 Variation in the coefficients used to calculate carbonation/enhanced weathering potential A value greater than zero increases the material carbon sequestration potential whereas a value less than zero reduces it. The value over the range of the dissolution environment pH is shown at standard temperature and pressure. Note for Na_2O and K_2O the coefficients ε and θ are = 1 for pH<12.

Supplementary Table 1 A summary of lime use in the US[1] and EU[12]			
**Product**	**US (1993-2015)**	**EU (2013)**	**Consequences of use**
Fertilizer/ agricultural lime	0.2	3	Reaction with CO_2 (and other soil acids for pH regulation)*
Glass	0.8	14	Reaction with silica and other cations (mainly Na) to produce a silicate glass. Potential for post use carbonation.
Paper and pulp	4.7		Reaction with Na_2CO_3 to produce $CaCO_3$ and NaOH in the Kraft Process**
Precipitated calcium carbonate	5.2		Reaction with CO_2*
Sugar refining	3.8		pH regulation and includes reaction with organic carbon compounds and impurities.**
Steel and iron	29.7	38	As a fluxing agent, ultimately ending up as a component of slag
Nonferrous metallurgy	5.7	-	
Asphalt	1.5	18	Reacts with the surface of silicate aggregates and organic acids in bitumen to stabilise an strengthen the asphalt.
Building uses + Other	1.9		As lime wash or lime mortars. Potential to carbonate as part of life-cycle*
Soil stabilization	6.5		Hydration (resulting in soil drying), and reaction with clays to produce calcium silicate hydrates. Some carbonation is also likely.*
Flue gas	17.2	16	Reaction with acid gasses to produce calcium sulphate minerals. Some carbonation is likely.
Sludge treatment:	1.7		Used to increase temperature and pH to disinfect prior to spreading on land. Potentially will carbonate in soils.*
Water treatment: AMD	0.8		Reaction with sulphuric acid in mine waters.
Drinking water	5.0		For pH regulation and precipitation of carbonate.*
Waste water	2.0		For neutralising acid waters.
* Activities involving the reaction of lime (quicklime or hydrated lime) with CO_2 to produce carbonates, these have been included in this model (approximately 20% of lime production). **Activities that promote reaction with CO_2 that was derived from biomass. There is potential to incorporate this into biomass energy carbon capture and storage, but has been excluded from this model.			

Supplementary Table 2 The values used for calculating the emissions intensity of alkaline materials

Material	Current emissions (kgCO_2 functional unit^{-1})	Future emissions (kgCO_2 functional unit^{-1})	Functional unit	functional units t^{-1} of material
Iron and steel slag	2200[10]	900 – 1400 (2030)<br>500 – 800 (2050)<br>200 – 500<br>(53[59†] - 2100[3*])	tonne steel	5.4[1]
Ordinary portland cement	800[10]	400 – 600 (2030)<br>200 – 400 (2050)<br>100 – 200 (2100)[3*]	tonne cement	1
Cement kiln dust				8.7[11]
Lime	785 (lime)[12]<br>~1000 (rounded)	80% reduction in current ~ 200	tonne lime	1
Ultrabasic mine tailings	2100 [13]	-	tonne nickel	0.004 - 0.012[14]
Coal ash	960[15]	96 – 125[15**]	MWh (coal)	21[15,16]
Biomass ash	18[17]	-600 to -1,400[17***]	MWh (bioenergy)	27[18–22]
Red mud	18,000[23]	3,600 (80% on current)[24]	tonne aluminium	0.3[1]

*using pre-commercial technologies – carbon capture and storage and decarbonised power
**includes oxyfuel.
***The range for biopower with carbon capture and storage
†Includes the integration of H_2 within the lifecycle of steel making

Supplementary Table 3 The regression parameters for aluminium, cement, lime, nickel, PGM and steel

Material	Saturation value (kg person^{-1}): a	b	Log m	r
Aluminium	21.5 ± 1.8	-4531.7 ± 841.7	-4.046	$-1.96 \times 10^{-4} \pm 6.80 \times 10^{-6}$
Cement	509.6 ± 26.4	-2562.5 ± 527.6	-4.000	$-6.08 \times 10^{-5} \pm 1.24 \times 10^{-6}$
Lime	62.9 ± 2.4	-1040.4 ± 233.1	-3.699	$-2.17 \times 10^{-4} \pm 4.33 \times 10^{-7}$
Nickel	2.7 ± 0.3	-3667.7 ± 1520.9	-4.222	$-2.91 \times 10^{-4} \pm 9.81 \times 10^{-7}$
PGM	$1.4 \times 10^{-3} \pm 1.5 \times 10^{-4}$	-18090.0 ± 2843.0	-4.523	$-6.85 \times 10^{-4} \pm 5.59 \times 10^{-6}$
Steel	463.1 ± 7.9	-2864.0 ± 117.9	-4.046	$-1.44 \times 10^{-4} \pm 1.21 \times 10^{-6}$

Supplementary Table 4 The parameters used in absorption model of CO_2 into concrete during service life

Type/ class	k_i (mm $yr^{-0.5}$)	di (mm)	Total thick. (mm)	Concrete use (%)	A_i (m^2)	V_i (m^3)	C_i (kg m^{-3})	W_i (kg)	V_i $yr^{-0.5}$ (m^3)	W_i (kg $yr^{-0.5}$)
Public	4.35	29.4	300	0.1	3.33	0.0980	217.4	2.13	0.015	0.315
Dam, power station, dock and infrastructure	3.66	24.7	225	0.1	4.44	0.1097	247.9	2.72	0.016	0.403
Railway, Road, tunnel, and bridge	4.05	27.3	160	0.3	6.25	0.1709	236.9	12.15	0.025	1.799
C+I	4.37	29.5	160	0.2	6.25	0.1843	217.5	8.02	0.027	1.188
Residential Buildings	4.15	28.1	200	0.3	5.00	0.1403	223.0	9.38	0.021	1.389

Parameters simplified from[2]

Supplementary Table 5 A summary of parameters used in Monte Carlo simulation							
**Variable**	**Distribution**	**Baseline**	**alpha**	**beta**	**Mode**	**Max**	**Min**
Clinker to cement rate	Weibull	0.9	25	91			
CaO Content in Clinker	Triangular	0.65			0.65	0.67	0.6
Proportion of Conversion Concrete	Weibull	0.8	25	86			
Proportion of Conversion Mortar	Weibull	0.8	20	92			
Service Life	Weibull	50	3	50			
Parameters simplified from[2]							

Supplementary Table 6 The carbonation reactions of minerals typically found in alkaline materials						
**Mineral**	**Formula**	**Material**	**ΔG$_f$ (kJ mol^{-1})**	**Carbonation reaction**	**ΔG$_f$ (kJ mol^{-1})**	**ΔG$_{f(n)}$ * (kJ mol^{-1})**
Anorthite[8]	$CaAl_2Si_2O_8$	S	-4007.9	$CaAl_2Si_2O_8 + CO_2 + 2H_2O \rightarrow CaCO_3 + Al_2Si_2O_5(OH)_4$	-59.5	-59.5
'Belite'/ Larnite[8]	Ca_2SiO_4	S,C	-2191.2	$Ca_2SiO_4 + 2CO_2 + 2H_2O \rightarrow 2CaCO_3 + H_4SiO_4$	-130.7	-65.4
Brucite[8]	$Mg(OH)_2$	S	-833.5	$Mg(OH)_2 + CO_2 \rightarrow MgCO_3 + H_2O$	-48.7	-48.7
Diopside	$MgCaSi_2O_6$	M	-3036.6	$MgCaSi_2O_6 + 2CO_2 + 4H_2O \rightarrow CaCO_3 + MgCO_3 + 2H_4SiO_4$	-19.9	-9.9
Forsterite[8]	Mg_2SiO_4	M, S	-2053.6	$Mg_2SiO_4 + 2CO_2 + 2H_2O \rightarrow 2MgCO_3 + H_4SiO_4$	-70.3	-35.1
Gehlenite[8]	$Ca_2Al_2SiO_7$	S	-3808.7	$Ca_2Al_2SiO_7 + 2CO_2 + 5H_2O \rightarrow 2CaCO_3 + 2Al(OH)_3 + H_4SiO_4$	-111.6	-55.8
Jennite-type (hydrated cement gel)[9]	$Ca_9Si_6O_{18}(OH)_6$ $8H_2O$	C	-13644.4	$Ca_9Si_6O_{18}(OH)_6 \cdot 8H_2O + 9CO_2 + H_2O \rightarrow 9CaCO_3 + 6H_4SiO_4$	-662.4	-73.6
Lime[8]	CaO	S, L, C	-603.1	$CaO + CO_2 \rightarrow CaCO_3$	-141.0	-141.0
Merwinite[8]	$Ca_3Mg(SiO_4)_2$	S	-4339.4	$Ca_3Mg(SiO_4)_2 + 4CO_2 + 4H_2O \rightarrow 3CaCO_3 + MgCO_3 + 2H_4SiO_4$	-205.3	-51.3
Periclase[8]	MgO	S	-569.2	$MgO + CO_2 \rightarrow MgCO_3$	-75.9	-75.9
Portlandite[8]	$Ca(OH)_2$	S, L, C	-898.4	$Ca(OH)_2 + CO_2 \rightarrow CaCO_3 + H_2O$	-82.8	-82.8
Rankinite[8]	$Ca_3Si_2O_7$	S, C	-3748.1	$Ca_3Si_2O_7 + 3CO_2 + 4H_2O \rightarrow 3CaCO_3 + 2H_4SiO_4$	-151.5	-50.5
Tobermorite-type (hydrated cement gel)[9]	$Ca_5Si_6O_{12}(OH)_{10}$ $3H_2O$	C	-10466.4	$Ca_5Si_6O_{12}(OH)_{10} \cdot 3H_2O + 5CO_2 + 4H_2O \rightarrow 5CaCO_3 + 6H_4SiO_4$	-626.8	-125.4
Tricalcium aluminate	$Ca_3Al_2(OH)_{12}$	R, C	-5019.3	$Ca_3Al_2(OH)_{12} + 3CO_2 \rightarrow 3CaCO_3 + 2Al(OH)_3 + 3H_2O$	-234.1	-78.0
Wollastonite[8]	$CaSiO_3$	S, C	-1549.9	$CaSiO_3 + CO_2 + 2H_2O \rightarrow CaCO_3 + H_4SiO_4$	-27.8	-27.8

ΔG$_f$ (kJ mol^{-1}) values for the other products and reactants were H_2O = -237.1, CO_2 = -384.4, H_4SiO_4 = -1307.8, $Al(OH)_3$ = -1154.9, $Al_2Si_2O_5(OH)_4$ = -3797.5, $CaCO_3$ = -1128.5, $MgCO_3$ = -1029.5, *S – slag, C – cement, L – lime, R – red mud, M – mine waste. **Gibbs free energy normalised to moles of CO_2 in the reaction.

**Supplementary Table 7 The major elemental composition of alkaline materials and their carbonation and enhanced weathering potential**								
**Material**	**CaO (%)**	**MgO (%)**	**Na_2O (%)**	**K_2O (%)**	**SO_3 (%)**	**P_2O_5 (%)**	**C_{pot} ($kgCO_2$ t^{-1})**	**E_{pot} ($kgCO_2$ t^{-1})**
Ash (biomass)[18] (n = 4)*	29.8 ± 8.2	7.0 ± 1.8	7.3 ± 3.7	15.0 ± 3.7	9.3 ± 4.8	11.8 ± 4.9	186.2 ± 126.1	461.6 ± 260.2
Ash (lignite)[16,25] (n=300)**	20.2 ± 1.0	5.8 ± 0.9	2.3 ± 0.9	0.4 ± 0.0	13.7 ± 1.8	0.1 ± 0.0	145.6 ± 27.9	245.5 ± 52.0
Ash (hard coal)[16] (n = 20)**	6.2 ± 0.4	1.3 ± 0.1	0.9 ± 0.1	1.4 ± 0.1	4.5 ± 0.3	0.5 ± 0.1	35.5 ± 5.7	73.0 ± 10.1
Cement[26] (n=1)	65.0	1.0	0.8	-	2.0	-	510	773
Cement kiln dust[27] (n = 63)	44.0 ± 1.0	1.6 ± 0.1	0.7 ± 0.1	4.0 ± 0.4	6.0 ± 0.5	-	330.0 ± 11.6	530.4 ± 21.4
Lime† (n = 14)	86.2 ± 1.4	9.2 ± 0.2	-	-	-	-	776.9 ± 12.9	1165 ± 19.4
Ni-laterite mine tailings[28] (n = 6)	0.2 ± 0.1	22.9 ± 9.4	-	-	-	-	251.0 ± 26.7	377.2 ± 40.1
Ni-sulphide mine tailings[29–31] (n = 31)	0.7 ± 0.1	34.2 ± 0.5	-	-	1.9 ± 0.2	-	367.5 ± 7.6	555.3 ± 11.7
PGM Ultrabasic mine waste[32] (n = 78)	5.4 ± 0.3	14.3 ± 0.6	1.2 ± 0.1	0.6 ± 0.1	0.3 ± 0.0	0.1 ± 0.0	196.6 ± 10.4	311.5 ± 17.2
Red mud[33,34] (n = 43)‡	5.7 ± 1.0	0.3	5.2 ± 0.6	0.4	0.1	-	46.8 ± 8.1	128.3 ± 18.1
Slag (Blast furnace)[35] (n = 11)	38.3 ± 0.8	11.6 ± 0.5	-	-	2.6 ± 0.2	0.1 ± 0.0	413.0 ± 12.5	619.5 ± 18.8
Slag (Steel)[35] (n = 45)	37.1 ± 1.0	9.1 ± 0.4	-	-	0.3 ± 0.0	0.7 ± 0.1	384.7 ± 13.4	577.1 ± 20.1

Note that the calculation procedure for the mineral carbonation (C_{pot}) and enhanced weathering (E_{pot}) potentials are presented in the online methods
*The biomass chemistry, ash, and energy content include data from a range of biomass sources including wood/woody biomass, herbaceous and agricultural residue, animal biomass, and marine algae.
**The average percentage ash content of lignite, hard coal, and biomass was 10.2 ± 1.2[16], 11.0 ± 4.2[16,25,36], and 6.9 ± 1.1[18–21,37] respectively, containing a higher heating value of 28, 14, and 19.1 ± 0.3 GJ t^{-1} respectively.
†Based on the relative production of high-calcium and dolomitic lime in the US between 2001 – 2014[38]
‡The red mud elemental composition considers untreated material, with larger concentrations of calcium typical in red mud stabilised by treatment with lime, to avoid double counting.

Supplementary Note 1

Table 1 in the manuscript presents the CO_2 emission intensities of alkaline materials, and their sequestration potentials through mineral carbonation and enhanced weathering. The method for calculating the potential (columns c and e, Table 1) are presented below. The current and future emissions intensities (columns a and b, Table 1) of the materials were calculated by taken known present or predicted future emissions for associated industries, and normalising them to the mass of the material (Supplementary Table 1). For instance, 2200 $kgCO_2$ are produced t^{-1} of steel, which may be reduced to 200 to 500 $kgCO_2$ with extensive mitigation (including decarbonised power and carbon capture and storage)[10]. Approximately 5 - 6 t steel is produced t^{-1} blast furnace slag. The emission intensity of slag is the product of these two numbers (12,000 $kgCO_2$ t^{-1} currently, or ~1,000 $kgCO_2$ t^{-1} with extensive mitigation).

Studies have shown that under controlled conditions it is possible to achieve levels of carbonation that approach the theoretical maximum (e.g., [39–44]), whereas other results have returned poor conversion (e.g., [45,46]). These are presented in column d in Table 1. The protocols for these experiments are not standardised, they do they test the sensitivity to a full range of control parameters, nor are they applicable to enhanced weathering estimates.

Supplementary Note 2

Approximately 500 Mt of slag is produced every year[1], estimates shown as shaded region annotated in Supplementary Figure 1. For every tonne of finished crude steel approximately 185 ± 5 and 117 ± 6 kg of blast furnace and steel slag are produced respectively[47]. Recycling waste metal (scrap) within the production of steel may result in a lower production of pig iron, which may be expected as economies move towards circular material life-cycles[48]. However, the average percentage of scrap in steel production has been largely constant (43 ± 0.7%) since the late 1960's[1], with trends decreasing (to 35% in 2014). The pig iron to steel production has also remained relatively constant (0.722 ± 0.002 t t^{-1}). It remains unclear how the global stock of steel (25 Gt currently in use[49]) will be reused or recycled over the next 100 years, how it will feed the material consumption of developed or developing economies, or the impact on slag production. These uncertainties have not been included within the model (for steel and other metals), and as such, we may overestimate the production of slag. The total carbonation potential of slag in 2100 (Supplementary Figure 1) may be between 320 $MtCO_2$ yr^{-1} (SSP3) and 870 $MtCO_2$ yr^{-1} (SSP2), and enhanced weathering potential between 480 and 1300 $MtCO_2$ yr^{-1}.

Supplementary Note 3

Heating limestone with clay or shale in a kiln at ~1500°C produces cement clinker, and cement kiln dust. Clinker is mixed at a ratio of ~9:1 with gypsum and other substitutes to create

cement, the ratio of which has reduced since the 1990's. Dust is produced as a by-product of typical kiln operation (cement kiln dust, CKD), in which a small selection of cement plants in the US produced 115 ± 17 kg t^{-1} clinker, 45 ± 10% of which is currently disposed of[11]. In Europe >80% of the cement produced is type CEM1 and CEM2, containing 100%, and more than 65%, portland cement (95% cement clinker + 5% gypsum) respectively. The balance is typically made up of blast furnace slag. In the US since the mid 1990's, cement clinker is 89 ± 0.2 % of total cement production[1]. China, the US, and Europe uses 70 ± 0.2 %. 87 ± 0.7%, and 72 ± 2% respectively of cement in concrete[2]. The balance being made up by use in mortar. Globally, it is estimated that 74 ± 1.5 % of cement is used in concrete[2], which was adopted and fixed within this model for future forecasts. The total amount of concrete or mortar produced in a given year (P) can be estimated using Eq1

$$P = P_{cem} \times R\left(\frac{\rho}{C}\right) \times 1000 \qquad \textbf{Eq1.}$$

where P_{cem} is the production of cement in a given year, R is the proportion of cement used in either concrete or mortar, ρ is the density or concrete or mortar (2.4 or 2.2 t m^{-3} respectively), and C is the cement content of concrete of mortar (294 ± 7.8 or 250 ± 6.6 kg m^{-3} respectively). Density values were assumed, cement contents were taken from[2].

We used a diffusion model (Eq2-4, described in detail in[2]) to estimate the absorption of CO_2 into cement and mortar during the life of a building.

$$d_{li} = k_{li} \times \sqrt{t} \qquad \textbf{Eq2.}$$

$$V_i = d_i \times A_i \qquad \textbf{Eq3.}$$

$$W_{li} = \sum V_i \times C_i \qquad \textbf{Eq4.}$$

where d_i is the absorption/carbonation depth of CO_2, and is related to the reaction time (t) with an empirically derived carbonation rate coefficient (k_i). The volume of carbonated material (V_i) is calculated by multiplying the depth of carbonation with the exposed area (A_i). k_i has been determined for a range of cement types/additives, surface coatings, strength characteristics, and ambient CO_2 concentrations. Given that future projections of the k_i controlling parameters

are uncertain, the published model[2] has been simplified taking globally representative values (Supplementary Table 4). An average mortar thickness of 20 mm was assumed, with a carbonation rate of 5 mm $yr^{-0.5}$. Values of CO_2 uptake were consistent with previously published values[2].

Demolition waste production was calculated by assuming a 50-year service life, and the CO_2 uptake potential was calculated by subtracting the proportion carbonated during the service life. Previous modelling work[2] does not consider the potential of carbonating demolition waste at the point of demolition, or its carbonation as a consequence of demolition practices[50]. We assume that 80% of remaining carbonation potential is used at the point of demolition which is consistent with ambient CO_2 capture in demolition waste[51]. We used a Monte Carlo simulation with 10,000 iterations for each scenario, with the parameters in Supplementary Table 5, to consider variations on these assumptions.

Using historical production estimates of cement, the results of CO_2 absorption into mortar and concrete during service life and following demolition between 1960 – 2014 are consistent with the results in Xi et al.,[2] (Supplementary Figure 2). Cement production may decrease to 3.5 Gt yr^{-1} (SSP5), or increase to 7.5 Gt yr^{-1} (SSP2) by 2100, and cement-based demolition waste (which also includes sand and aggregate, 90-85 % by mass) may increase to 20 – 40 Gt yr^{-1} (Figure 2). Forecasts for 2100 CO_2 absorption into cement during the life cycle or carbonation following demolition range between 1.5 $GtCO_2$ yr^{-1} (SSP5) and 3.5 CO_2 yr^{-1} (SSP2). If enhanced weathering is promoted in the demolition waste, the carbon sequestration potential may be between 2.2 and 4.7 $GtCO_2$ yr^{-1} respectively.

Supplementary Note 4

Approximately 300 million tonnes of lime are produced annually[1]. In the US, 41 ± 1 % was used in steel manufacturing, 27 ± 0.6 % was used in chemical and industrial applications, 8 ± 0.4 % was used in construction, and 22 ± 0.9% was used in environmental applications between 1975 and 2003[13]. A similar distribution is found in the EU (Supplementary Table 1[12]).

The recarbonation of lime is a thermodynamically likely consequence of its use or life-cycle (see Supplementary Table 6), particularly in environmental applications. Some applications explicitly involve reaction with CO_2 (e.g., recovery of NaOH in the Kraft Process of paper manufacturing), whereas others use lime to neutralise sulphuric acid (acid mine drainage, or flue gas desulphurisation). Approximately 20% of lime production has a reaction with CO_2 during its life-cycle (which has been used in this model), 8.5% of lime is reacted with CO_2 from biomass (in the Kraft process and sugar refining, this has not been included in this model), and a further 2.3 % of lime may be available for carbonation at the end of lime of its product (glass and asphalt), however this has not been included. Lime use in the steel industry has not been included (to avoid double counting with the carbonation potential of slag). We have not included lime that is reacted with stronger acids in the model (acid mine drainage treatment, or flue gas desulphurisation), however this may represent an avoided emission has acid mine water or acid rain is buffered in the environment by the carbonate system. The carbonation potential of lime is between 60 and 143 $MtCO_2\ yr^{-1}$ by 2100. While some calcium from dissolved lime may remain in solution, due to the difficulty in separating these reactions from precipitated carbonate, we assume no enhanced weathering in lime.

Supplementary Note 5

Approximately 600 Mt of ash are produced every year from coal combustion, equating to between 3.6 and 7.9 kg of ash GJ^{-1} of primary energy. Between 1998 and 2013 coal use was 90.2 ± 1.0 % and 9.8 ± 0.3 % ‘hard coal’ (anthracite and bituminous) or lignite respectively [38]. Lignite use decreased over that period to 8.5%. Future production of ash is heavily dependent on transformations within the energy system. Baseline SSPs predict increases in coal use, whereas decreases are predicted in RCPs 2.6, 3.4, and 4.5. For future forecasts, we assume that the proportion of lignite in the coal mix to decrease to zero in all scenarios. While this may be unlikely in scenarios that predict increases in coal use, it is a conservative assumption given that the carbonation potential of hard coal is approximately a quarter that of lignite (Supplementary Table 7). Coal ash production may remain similar to current values (SSP1)

or increase to 4.2 Gt yr^{-1} (SSP5) by 2100. RCP 2.6 and 3.4 largely predict decreases to 140 – 270 Mt yr^{-1} (Supplementary Figure 3). The carbonation potential of coal ash may be between 5 and 150 $MtCO_2$ yr^{-1}, and the enhanced weathering potential may be between 10 – 305 $MtCO_2$ yr^{-1}.

Oxidising organic carbon in biomass to produce energy results in a by-product relatively enriched in the non-oxidized products (Ca, Mg, Si, Al, etc.), regardless of the method of energy conversion. Conceptually the production of ash from complete biomass combustion provides the simplest method of calculating carbonation potential of these by-products. The proportion and chemistry of ash varies with biomass source (0.2 – 39 %), with an average of 6.9 ± 1.1 % (3.6 ± 0.6 kg GJ^{-1})[16,25,36] and the average major elemental composition provided in Supplementary Table 7. These values are indicative of the range of sources, and not a prediction of future biomass mix. Future production of ash (Supplementary Figure 3) may be as large as 1.2 Gt yr^{-1} (RCP 2.6), or 230 Mt yr^{-1} (SSP2). The carbonation potential of biomass ash is between 44 and 229 $MtCO_2$ yr^{-1}. Alternatively, the enhanced weathering potential is between 110 and 569 $MtCO_2$ yr^{-1}.

Supplementary Note 6

The production of 1 tonne of aluminium produces 3.45 ± 0.04 tonnes of red mud (also known as 'bauxite residues')[52]. Approximately 120 Mt of red mud are produced annually (with a global stock of approximately 3 Gt)[4]. The acid neutralising capacity of red mud is approximately 10 moles kg^{-1} [53], or notionally 44-66 $kgCO_2$ t^{-1} if neutralising carbonic acid, which is derived from the reaction of the NaOH rich liquor with CO_2 (Eq5)

$$NaOH_{(aq)} + CO_2 \rightarrow xNaHCO_{3\,(aq)} + yNa_2CO_{3\,(aq)} \qquad \text{Eq5.}$$

The neutralising capacity of red mud equates to approximately 40 kgNaOH t^{-1}, or approximately ~5 Mt of sodium hydroxide. This is consistent with NaOH use in the Bayer Process (0.09 t of NaOH t^{-1} of aluminium, or 4.5 Mt NaOH[54]). However, to avoid double counting with the sodium content of solid red mud (which may have precipitated from solution

during drying prior to analysis), the carbonation potential of red mud was calculated from the major elemental composition alone (Supplementary Table 7). Red mud production is predicted to increase to between 360 Mt yr^{-1} (SSP3) and 1.2 Gt yr^{-1} (SSP2, Supplementary Figure 4), with a carbonation potential of 17-55 $MtCO_2$ yr^{-1}. The enhanced weathering potential of red mud, given the contribution from Na, may be greater (45 – 150 $MtCO_2$ yr^{-1})

Supplementary Note 7

The extraction of some metals produces overburden or tailings material that is rich in ultrabasic rocks. As the concentration of the metal in the ore is less than a few percent for most metals, the waste rock production can be substantial. The most promising mining waste is generated from metals contained in ultrabasic host deposits. Previous work has investigated material associated with chrysotile asbestos ($Mg_3(Si_2O_5)(OH)_4$) mining, in which carbonation has been detected in the drainage waters, attributed to weathering of the accessory mineral brucite. It remains unclear, even under favourable conditions, that the entire rock mass would weather sufficiently rapidly[55]. While historically important, asbestos mining has limited future potential, and has not been considered within future production estimates. Other mine waste has also been considered for use in CO_2 capture including those associated with nickel, platinum group metals, and diamonds. The first two represent the largest potential, with current waste rock and tailings production of 100-200 Mt yr^{-1} (Ni-laterites and platinum group metals; Supplementary Figure 5). This may increase to several hundreds of Mt yr^{-1} to 2 Gt yr^{-1} by 2100.

Supplementary Note 8

We used a non-linear least squares regression through 1960 – 2014 national production data[1] (or regional aggregates for aluminium[52]) to derive saturation values of $capita^{-1}$ consumption (see Figure 1 and the online methods). As <5% of cement and lime are traded internationally, national production data were used for simplicity. Historical GDP data[56] were normalised to 2014 US$ values, and divided by historical population to derive GDP $capita^{-1}$. The regression procedure is described in the online methods, and the parameters and their uncertainties are shown in Supplementary Table 3.

Aluminium consumption data were derived from[52] and includes information for China, Japan, Europe (Albania, Austria, Belgium, Bosnia Herzegovina, Bulgaria Belarus, Croatia, Czechia, Denmark, Finland, France, Germany, Greece, Hungary, Italy, Latvia, Luxembourg, Rep. of Moldova, Montenegro, Netherlands, Norway, Poland, Portugal, Romania, Serbia, Slovakia, Slovenia, Spain, Sweden, Switzerland, Turkey, Ukraine, TFYR of Macedonia, United Kingdom), Latin/South America (Argentina , Brazil , Venezuela) , Middle East (Iran, Oman, Qatar, Saudi Arabia, United Arab Emirates), North America (Canada, Mexico, the United States), 'Other Asia' (India , Indonesia , Malaysia, Pakistan , Rep. of Korea , Singapore , Thailand , Viet Nam), and 'Other Producing Countries' (Azerbaijan, Egypt, Ghana, Kazakhstan, New Zealand, Nigeria, Russian Federation, South Africa, Zimbabwe).

Cement production data were derived from a compilation of production statistics from United States Geological Survey (USGS) Mineral Yearbooks[1] from 1960 to 2015 and includes information for Argentina, Australia, Brazil, Canada, China, the European Union, Indonesia, India, Japan, Mexico, Middle East, New Zealand, Rep. of Korea, Russia, South Africa, Thailand, and the United States. These countries have produced 76 ± 14 % (s.d.) of global annual cement since 1960.

Steel consumption data was compiled from USGS Mineral Yearbook[1] production statistics from 1967 to 2015 and includes information for Albania, Algeria, Argentina, Australia, Austria, Azerbaijan, Bangladesh, Belgium and Luxemburg, Bosnia-Herzegovina, Brazil, Bulgaria, Byelorussia, Canada, Chile, China, Colombia, Croatia, Cuba , Czech Republic, Denmark, Ecuador, Egypt, El Salvador, Finland, France, Germany, Ghana, Greece, Guatemala, Hungary, India, Indonesia, Iran, Israel, Italy, Japan, Jordan, Kazakhstan, Kenya, Latvia, Libya, TFYR of Macedonia, Malaysia, Mexico, Moldova, Mongolia, Montenegro, Morocco , Myanmar, Netherlands, New Zealand, Nigeria, Norway, Oman, Pakistan, Paraguay, Peru, Philippines, Poland, Portugal, Qatar, Rep. of Korea, Romania, Russia, Saudi Arabia, Serbia, Singapore, Slovak Republic, Slovenia, South Africa, Spain, Sri Lanka, Sweden, Switzerland, Syria, Thailand, Trinidad and Tobago, Tunisia, Turkey, Uganda, Ukraine, United Arab Emirates,

United Kingdom, United States, Uruguay, Uzbekistan, Venezuela, Viet Nam, and Zimbabwe. These countries equate to 95 ± 13 (s.d.)% of the global steel consumption since 1967.

Global lime production data were derived from a compilation of production statistics from USGS Mineral Yearbooks[1] from 1990 to 2015 and include information for Australia, Brazil, Canada, China, European Union, India, Japan, Mexico, New Zealand, Russia, South Africa, and the United States. These countries equate to 62 ± 22 (s.d.)% of the global lime production since 1990

Nickel production and consumption data were derived from[57] and includes information for Australia, Austria, Belgium and Luxemburg, Brazil, Canada, China, Colombia, Cuba, Germany, Dominican Republic, Egypt, Spain, Finland, France, United Kingdom, Hong Kong, Indonesia, India, Italy, Japan, South Korea, Netherlands, Norway, Russia, Singapore, Sweden, Thailand, Turkey, Taiwan, the United States, Viet Nam, and Zimbabwe. These equate to >90% of global production since 1995.

There is less available national data on platinum group metals (PGM) consumption. Here we use data from[58], which includes information for Japan, The European Union, and North America. These regions consumed the equivalent of 133% of global production between 1987 and 2002, the discrepancy is potentially the result of 20-30% of PGM sourced through recycling[58].

For the years in which production or consumption information was available, these data were compiled with population and 2014 normalised gross domestic product data from[45]

Supplementary References

1. USGS. *USGS Minerals Yearbook*. (U.S. Department of the Interior and U.S. Geological Survey, 2016).
2. Xi, F. et al. Substantial global carbon uptake by cement carbonation. *Nat. Geosci.* **9**, 880 (2016).

3. Manz, O. E. Worldwide production of coal ash and utilization in concrete and other products. *Fuel* **76**, 691–696 (1997).
4. Power, G., Gräfe, M. & Klauber, C. Bauxite residue issues: I. Current management, disposal and storage practices. *Hydrometallurgy* **108**, 33–45 (2011).
5. Riahi, K. et al. The Shared Socioeconomic Pathways and their energy, land use, and greenhouse gas emissions implications: An overview. *Glob. Environ. Change* **42**, 153–168 (2017).
6. O'Neill, B. C. et al. A new scenario framework for climate change research: the concept of shared socioeconomic pathways. *Clim. Change* **122**, 387–400 (2014).
7. Parkurst, D. L. & Appelo, C. User's guide to PHREEQC (Version 2): A computer program for speciation, batch-reaction, onedimensional transport, and inverse geochemical calculations. (1999).
8. Robie, R. A. & Hemingway, B. S. *Thermodynamic properties of minerals and related substances at 298.15 K and 1 bar (105 Pascals) pressure and at higher temperatures*. **2131**, (US Government Printing Office, 1995).
9. Matschei, T., Lothenbach, B. & Glasser, F. P. Thermodynamic properties of Portland cement hydrates in the system CaO–Al2O3–SiO2–CaSO4–CaCO3–H2O. *Cem. Concr. Res.* **37**, 1379–1410 (2007).
10. IPCC. Technology-specific Cost and Performance Parameters. in *Climate Change 2014: Mitigation of Climate Change*: Working Group III Contribution to the IPCC Fifth Assessment Report (ed. IPCC) 1329–1356 (Cambridge University Press, 2015). doi:10.1017/CBO9781107415416.025
11. EPA. *Management Standards Proposed for Cement Kiln Dust Waste.* (Environmental Protection Agency, 1999).
12. EuLA. *A Competitive and Efficient Lime Industry*. Prepared by Ecofys. (European Lime Association, 2014).
13. Farrell, M. Carbon emissions from base metal mine sites. *Min. Eng.* 29 (2009).

14. Mudd, G. M. & Jowitt, S. M. A Detailed Assessment of Global Nickel Resource Trends and Endowments. *Econ. Geol.* **109**, 1813–1841 (2014).
15. Krey, V. et al. Annex 2-Metrics and methodology. In: *Climate Change 2014: Mitigation of Climate Change. IPCC Working Group III Contribution to AR5.* Cambridge University Press. (2014).
16. Selvig, W. A. & Gibson, F. H. *Analyses of ash from coals of the United States.* (U.S. G.P.O., 1945).
17. Edenhofer, O. et al. *IPCC special report on renewable energy sources and climate change mitigation.* Prep. Work. Group III Intergov. Panel Clim. Change Camb. Univ. Press Camb. UK (2011).
18. Vassilev, S. V., Baxter, D., Andersen, L. K. & Vassileva, C. G. An overview of the composition and application of biomass ash. Part 1. Phase–mineral and chemical composition and classification. *Fuel* **105**, 40–76 (2013).
19. McKendry, P. Energy production from biomass (part 1): overview of biomass. *Rev. Issue* **83**, 37–46 (2002).
20. de Jong, W. *Biomass Composition, Properties, and Characterization. in Biomass as a Sustainable Energy Source for the Future* 36–68 (Wiley-Blackwell, 2014). doi:10.1002/9781118916643.ch2
22. Demirbas, A. Trace Element Concentrations in Ashes from Various Types of Lichen Biomass Species. *Energy Sources* **26**, 499–506 (2004).
22. Lizotte, P.-L., Savoie, P. & De Champlain, A. Ash content and calorific energy of corn stover components in Eastern Canada. *Energies* **8**, 4827–4838 (2015).
23. Nunez, P. & Jones, S. Cradle to gate: life cycle impact of primary aluminium production. *Int. J. Life Cycle Assess.* **21**, 1594–1604 (2016).
24. Liu, G., Bangs, C. E. & Müller, D. B. Unearthing Potentials for Decarbonizing the U.S. Aluminum Cycle. *Environ. Sci. Technol.* **45**, 9515–9522 (2011).

25. Dai, S. et al. Geochemistry of trace elements in Chinese coals: A review of abundances, genetic types, impacts on human health, and industrial utilization. *Miner. Trace Elem. Coal* **94**, 3–21 (2012).

26. Van Oss, H. G. & Padovani, A. C. Cement manufacture and the environment part II: environmental challenges and opportunities. *J. Ind. Ecol.* **7**, 93–126 (2003).

27. Sreekrishnavilasam, A., King, S. & Santagata, M. Characterization of fresh and landfilled cement kiln dust for reuse in construction applications. *Geoenvironmental Eng.* **85**, 165–173 (2006).

28. Marsh, E., Anderson, E., Gray, F., US Department of the Interior & US Geological Survey. *Nickel–cobalt laterites—A deposit model. Mineral deposit models for resource assessment*: US Geological Survey Scientific Investigations Report 2010-5070 (2013).

29. Hamilton, J. L. et al. Fate of transition metals during passive carbonation of ultramafic mine tailings via air capture with potential for metal resource recovery. *Int. J. Greenh. Gas Control* **71**, 155–167 (2018).

30. Kandji, E. H. B., Plante, B., Bussière, B., Beaudoin, G. & Dupont, P.-P. Kinetic testing to evaluate the mineral carbonation and metal leaching potential of ultramafic tailings: Case study of the Dumont Nickel Project, Amos, Québec. *Appl. Geochem.* **84**, 262–276 (2017).

31. Heikkinen, P. M. & Räisänen, M. L. Mineralogical and geochemical alteration of Hitura sulphide mine tailings with emphasis on nickel mobility and retention. *J. Geochem. Explor.* **97**, 1–20 (2008).

32. Vogeli, J., Reid, D.L., Becker, M., Broadhurst, J. & Franzidis, J.-P. Investigation of the potential for mineral carbonation of PGM tailings in South Africa. *Miner. Eng.* **24** 1348-1356 (2011).

33. Gräfe, M., Power, G. & Klauber, C. Bauxite residue issues: III. Alkalinity and associated chemistry. *Hydrometallurgy* **108**, 60–79 (2011).

34. Arslan, S., Demir, G. K., Celikel, B., Baygul, M. & Suarez, C. E. ETI Aluminum Red Mud Characterization and Processing. in *Light Metals 2012* 81–85 (Wiley-Blackwell, 2012). doi:10.1002/9781118359259.ch15
35. Proctor, D. M. et al. Physical and Chemical Characteristics of Blast Furnace, Basic Oxygen Furnace, and Electric Arc Furnace Steel Industry Slags. *Environ. Sci. Technol.* **34**, 1576–1582 (2000).
36. Koukouzas, N. K., Zeng, R., Perdikatsis, V., Xu, W. & Kakaras, E. K. Mineralogy and geochemistry of Greek and Chinese coal fly ash. Spec. Issue 2005 *World Coal Ash Conf.* **85**, 2301–2309 (2006).
37. Zanzi, R. Pyrolysis of biomass. Rapid pyrolysis at high temperature. Slow pyrolysis for active carbon preparation. (2001).
38. USGS. *USGS Minerals Yearbook*. (U.S. Department of the Interior and U.S. Geological Survey, 2015).
39. Chang, E.-E., Chen, C.-H., Chen, Y.-H., Pan, S.-Y. & Chiang, P.-C. Performance evaluation for carbonation of steel-making slags in a slurry reactor. *J. Hazard. Mater.* **186**, 558–564 (2011).
40. Fernández Bertos, M., Simons, S. J. R., Hills, C. D. & Carey, P. J. A review of accelerated carbonation technology in the treatment of cement-based materials and sequestration of CO2. *J. Hazard. Mater*. **112**, 193–205 (2004).
41. Gunning, P. J., Hills, C. D. & Carey, P. J. Accelerated carbonation treatment of industrial wastes. *Waste Manag.* **30**, 1081–1090 (2010).
42. Montes-Hernandez, G., Pérez-López, R., Renard, F., Nieto, J. M. & Charlet, L. Mineral sequestration of CO2 by aqueous carbonation of coal combustion fly-ash. *J. Hazard. Mater.* **161**, 1347–1354 (2009).
43. Huijgen, W. J. J. & Comans, R. N. J. Mineral CO2 Sequestration by Steel Slag Carbonation. *Environ. Sci. Technol.* **39**, 9676–9682 (2005).

44. Eloneva, S., Teir, S., Salminen, J., Fogelholm, C.-J. & Zevenhoven, R. Steel Converter Slag as a Raw Material for Precipitation of Pure Calcium Carbonate. *Ind. Eng. Chem. Res.* **47**, 7104–7111 (2008).
45. Yadav, V. S. et al. Sequestration of carbon dioxide (CO2) using red mud. *J. Hazard. Mater.* **176**, 1044–1050 (2010).
46. van Zomeren, A., van der Laan, S. R., Kobesen, H. B. A., Huijgen, W. J. J. & Comans, R. N. J. Changes in mineralogical and leaching properties of converter steel slag resulting from accelerated carbonation at low CO2 pressure. *Waste Manag.* **31**, 2236–2244 (2011).
47. U.S. Geological Survey. *Mineral commodity summaries 2018*. 200 (2018).
48. Neelis, M. & Patel, M. Long-term production, energy consumption, and CO2 emission scenarios for the worldwide iron and steel industry. (Copernicus Institute, Utrecht University).
49. Krausmann, F. et al. Global socioeconomic material stocks rise 23-fold over the 20th century and require half of annual resource use. *Proc. Natl. Acad. Sci.* **114**, 1880 (2017).
50. Washbourne, C.-L., Renforth, P. & Manning, D. A. C. Investigating carbonate formation in urban soils as a method for capture and storage of atmospheric carbon. *Sci. Total Environ.* **431**, 166–175 (2012).
51. Washbourne, C.-L., Lopez-Capel, E., Renforth, P., Ascough, P. L. & Manning, D. A. C. Rapid Removal of Atmospheric CO2 by Urban Soils. *Environ. Sci. Technol.* **49**, 5434–5440 (2015).
52. Bertram, M. et al. A regionally-linked, dynamic material flow modelling tool for rolled, extruded and cast aluminium products. *Resour. Conserv. Recycl.* **125**, 48–69 (2017).
53. Renforth, P. et al. Contaminant mobility and carbon sequestration downstream of the Ajka (Hungary) red mud spill: The effects of gypsum dosing. *Sci. Tot. Environ.* **421–422**, 253–259 (2012).

54. Dewan, M., Rhamdhani, M., Brooks, G., Monaghan, B. & Prentice, L. Alternative Al production methods: Part 2–thermodynamic analyses of indirect carbothermal routes. *Miner. Process. Extr. Metall.* **122**, 113–121 (2013).

55. Harrison, A. L., Power, I. M. & Dipple, G. M. Accelerated Carbonation of Brucite in Mine Tailings for Carbon Sequestration. *Environ. Sci. Technol.* **47**, 126–134 (2013).

56. World Bank. World Development Indicators: GDP (current US$). Available https://data.worldbank.org/. (2018).

57. Nakajima, K. et al. Global distribution of material consumption: Nickel, copper, and iron. *Resour. Conserv. Recycl.* **133**, 369–374 (2018).

58. Wilburn, D. R. & Bleiwas, D. I. Platinum-group metals—world supply and demand. US *Geol. Surv. Open-File Rep.* **1224**, 2004–1224 (2004).

59. Vogl, V., Åhman, M. & Nilsson, L. J. Assessment of hydrogen direct reduction for fossil-free steelmaking. *J. Clean. Prod.* **203**, 736–745 (2018).
