# Supplementary material for: The negative emission potential of alkaline materials
Source: Nat Commun. 2019 Mar 28;10:1401. doi: 10.1038/s41467-019-09475-5 (PMC6438983; doi:10.1038/s41467-019-09475-5)
Supplement: Supplementary file 3 — Description of Additional Supplementary Files [file 41467_2019_9475_MOESM3_ESM.pdf]

Description of Additional Supplementary Information

File Name: Supplementary Data 1
Description: Total CO_2 draw-down for alkaline materials: Mineral Carbonation

File Name: Supplementary Data 2
Description: Total CO_2 draw-down for alkaline materials: Enhanced Weathering

File Name: Supplementary Data 3
Description: Aluminium and red mud production

File Name: Supplementary Data 4
Description: Cement and by-products production

File Name: Supplementary Data 5
Description: Biomass, coal and ash production

File Name: Supplementary Data 6
Description: Steel and slag production

File Name: Supplementary Data 7
Description: Lime production

File Name: Supplementary Data 8
Description: Nickel and nickel tailings production

File Name: Supplementary Data 9
Description: PGM and PGM tailings production
